# Role of Exogenous Hsp72 on Liver Dysfunction during Sepsis

**DOI:** 10.1155/2015/508101

**Published:** 2015-06-29

**Authors:** Tsen-Ni Tsai, Jia-Jing Ho, Maw-Shung Liu, Tzu-Ying Lee, Mei-Chin Lu, Chia-Jen Liu, Li-Ju Huang, Sheng-I Lue, Rei-Chen Yang

**Affiliations:** ^1^Graduate Institute of Medicine, Kaohsiung Medical University, Kaohsiung 807, Taiwan; ^2^Department of Physiology, College of Medicine, Kaohsiung Medical University, Kaohsiung 807, Taiwan; ^3^Graduate Institute of Marine Biotechnology, National Dong Hwa University, Pingtung 97401, Taiwan; ^4^Center of Teaching and Research, Kaohsiung Municipal Ta-Tung Hospital, Kaohsiung Medical University Hospital, Kaohsiung 80145, Taiwan; ^5^Department of Pediatrics, Kaohsiung Medical University Hospital, Kaohsiung 807, Taiwan; ^6^Department of Pediatrics, Changhua Christian Hospital, Changhua 500, Taiwan

## Abstract

This study examined the role of exogenous heat shock protein 72 (Hsp72) in reversing sepsis-induced liver dysfunction. Sepsis was induced by cecal ligation and puncture. Liver function was determined on the basis of the enzymatic activities of serum glutamate oxaloacetate transaminase (GOT) and glutamate pyruvate transaminase (GPT). Apoptosis was determined using terminal deoxynucleotidyl transferase dUTP nick end labeling staining. B-cell lymphoma 2 (Bcl-2), Bcl-2-associated X protein (Bax), cleaved caspase-3 and caspase-9, and cleaved poly (ADP-ribose) polymerase (PARP) protein expressions were analyzed using Western blotting. Results showed GOT and GPT levels increased during sepsis, and levels were restored following the administration of human recombinant Hsp72 (rhHsp72). Increased liver tissue apoptosis was observed during sepsis, and normal apoptosis resumed on rhHsp72 administration. The Bcl-2/Bax ratio, cleaved caspase-3, caspase-9, and PARP protein expressions in the liver tissues were upregulated during sepsis and normalized after rhHsp72 treatment. We conclude that, during sepsis, exogenous Hsp72 restored liver dysfunction by inhibiting apoptosis via the mitochondria-initiated caspase pathway.

## 1. Introduction

Sepsis, the most prevalent cause of death in intensive care units, annually affects over 500,000 patients in the United States [1]. Despite advances in treatment and supportive care, the mortality rate continues to exceed 20% [2]. Sepsis bacteria invade the blood stream and release various microbial products. In response, the host cells initiate innate immune responses [3], which cause adverse metabolic alterations leading to systemic inflammatory responses, tissue dysfunction, and eventually multiple organ failure (MOF) [4]. The liver-gut axis was considered responsible for the inflammatory reaction and the subsequent MOF [5]. During sepsis, the liver participates in the defense action and tissue repair in the host through hepatic cell cross-talk, controlling most of the coagulatory and inflammatory processes [6]. The incidence of sepsis-associated episodes ranges from 34% to 46% for liver dysfunction and from 1.3% to 22% for liver failure [7]. An animal study revealed that inhibiting hepatocyte apoptosis reverses sepsis-induced liver dysfunction [8]. Furthermore, treating liver injury and restoring liver function lowers the morbidity and mortality in patients with sepsis [7]. Our previous study revealed that heat shock prevents sepsis-induced liver apoptosis via increased expression of heat shock protein 72 (Hsp72/Hp70) [9]. Several studies have indicated that Hsp72 overexpression protects cells from apoptosis because of the increased Bcl-2/Bax ratio [9, 10]. Hsp72/Hp70, a molecular chaperone induced in vivo by stress, exhibits anti-inflammatory [11] and antiapoptotic effects [12].

In addition to its in vivo action, the extracellular Hsp72 (eHsp72) protein is crucial in protecting against cell damage. The intracellular Hsp72 protein is actively released into the extracellular milieu in response to various stimuli [13–15]. Clinically, serum Hsp72 levels are elevated in patients with infection [16], diabetes [17], ischemia and reperfusion [18], trauma [19], and sepsis [20]. Furthermore, increase in the serum Hsp72/Hsp70 levels and disease severity as well as event outcomes in critically ill patients has been positively correlated [21, 22]. An animal study demonstrated that pretreatment using recombinant Hsp70 decreases endotoxin-shock induced mortality [23]. In vitro cell culture experiments showed that exogenous Hsp72 confers apoptosis protection [24] and decreases inflammatory cytokine production [25, 26]. Takemoto et al. reported that Hsp72 accumulates mainly in the liver after intravenous or subcutaneous injection of recombinant Hsp72 [27]. These observations demonstrate that exogenous Hsp72 protects cells against damage under various pathological conditions, including sepsis [25]. Despite the numerous evidences for the protective role of exogenous Hsp72, the underlying protection mechanism remains unclear. Therefore, we investigated the role of rhHsp72 in improving liver function during sepsis through apoptosis inhibition.

## 2. Materials and Methods

### 2.1. Materials

Human recombinant Hsp72 (low endotoxin) was obtained from Enzo Life Science (Farmingdale, NY, USA). Rabbit anti-B-cell lymphoma 2 (Bcl-2), anticleaved caspase-3, and anticleaved caspase-9 antibodies were obtained from Cell Signaling Technology (Danvers, MA, USA). Goat anti-rabbit immunoglobulin G [28] was obtained from Jackson ImmunoResearch Laboratories (West Grove, PA, USA), goat anti-mouse IgG was purchased from Abcam (Cambridge, MA, USA), and rabbit anti-glyceraldehyde 3-phosphate dehydrogenase (GAPDH) monoclonal and mouse anti-Bcl-2-associated X protein (Bax) polyclonal antibodies were obtained from Santa Cruz Biotechnology, Inc. (Delaware Avenue, California, USA). Rabbit anticleaved poly (ADP-ribose) polymerase (PARP-1) polyclonal antibody was obtained from Bioworld Technology, Inc. (Minneapolis, MN, USA). Enhanced chemiluminescent detection system was obtained from Pharmacia Biotech (Välinge, Sweden). In situ cell death detection kit and horseradish peroxidase (POD) were purchased from Roche Applied Science (Mannheim, Germany). Other chemicals and reagents were of analytical grade.

### 2.2. rhHsp72 Administration in Animal Models

All experiments were performed according to the guidelines of the National Institutes of Health for the Care and Use of Laboratory Animals. The experimental protocols were approved by Kaohsiung Medical University Committee for the Use of Experimental Animals. Male Sprague-Dawley rats (weight, 165–180 g; BioLasco Taiwan Co., Ltd) were used as experimental animals. Three groups containing 12 rats each were formed: sham, cecal ligation, and puncture (CLP) and CLP + rhHsp72. Sepsis was induced using CLP [29] with minor modifications. Laparotomy was performed under the influence of isoflurane anesthesia, and the cecum was ligated below the ileocecal valve and punctured twice using an 18-gauge needle. Subsequently, the bowel was returned to the peritoneal cavity, and the abdominal incision was closed in two layers. Control animals underwent a sham surgery in which laparotomy was performed but the cecum was left untouched without ligation and perforation. All animals were subcutaneously administered 4 mL/100 g body weight of normal saline after the surgery and once a day thereafter. After recovery, the animals were returned to their holding room and observed every 2 h until they reached the experimental endpoints. Human recombinant Hsp72 (20 *μ*g/kg) was administered subcutaneously immediately after CLP, and blood samples and liver tissues were collected 18 and 24 h after CLP for biochemical and histopathological analyses.

### 2.3. Determination of Liver Function

Liver function was determined on the basis of the enzymatic analysis of glutamate oxaloacetate transaminase/aspartate transaminase (GOT/AST) and glutamate pyruvate transaminase/alanine aminotransferase (GPT/ALT) [30]. GOT and GPT activities were assayed using a biochemical blood analyzer (DRI-CHEM 3500s, Fujifilm, Japan).

### 2.4. Analysis of Liver Tissue Apoptosis

Liver tissues were fixed in 4% paraformaldehyde for 24 h followed by dehydration in graded percentages of alcohol. Paraffin-embedded liver tissues were sectioned at 5 *μ*m intervals, and apoptosis was determined using the terminal deoxynucleotidyl transferase dUTP nick end labeling (TUNEL) assay according to the manufacturer's instructions. These tissue sections were incubated in 10 *μ*g/mL proteinase K in 0.1 M PBS (pH 7.4) for 30 min at 37°C after deparaffinization. Subsequently, the tissue sections were immersed in 0.1 M citrate buffer (pH 6) under microwave irradiation and then incubated in the TUNEL reaction mixture containing terminal deoxynucleotidyl transferase for 1 h at 37°C. After washing with PBS, the sections were incubated with POD for 30 min followed by DAB subtracts. Next, the tissue sections were mounted under glass cover slides and imaged through optical microscopy (Eclipse 80i, Nikon, Japan). Total nucleoli were identified using hematoxylin staining. Percentages of TUNEL-positive cells, indicative of DNA damage, were calculated as the ratio of the number of TUNEL-positive cells to the total number of nucleoli.

### 2.5. Western Blot Analysis of Protein Expression of Bcl-2, Bax, Cleaved Caspase-3, Cleaved Caspase-9, and Cleaved PARP

Liver homogenate containing 30 *μ*g of tissue was subjected to protein denaturation and sodium dodecyl sulfate-polyacrylamide gel electrophoresis [31]. The separated proteins were transferred onto polyvinylidene difluoride membranes through electroblotting for 1 h (100 V). After blocking with 5% nonfat milk in Tris-buffer saline, the membranes were incubated overnight in primary antibody at 4°C. Subsequently, the membranes were incubated in a secondary antibody at room temperature for 1 h. Protein bands were visualized using an enhanced chemiluminescence kit (Amersham) and Fuji medical X-ray films, and the relative densities were quantified.

### 2.6. Statistical Analyses

All values were expressed as mean ± standard error of the mean (SE). Data analyses and evaluation of statistical significance between the two groups of parameters were performed using ANOVA and Tukey's test; *p* < 0.05 was considered statistically significant.

## 3. Results 

### 3.1. Effects of rhHsp72 on Liver Function during Sepsis

**Figure 1:**
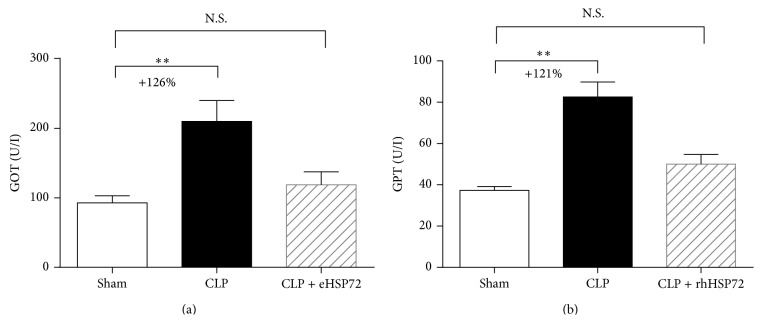
Effects of rhHsp72 on liver function. Serum samples were harvested 18 h after CLP and sham surgery. Liver function was determined through enzymatic analysis of glutamate oxaloacetate transaminase (GOT) (a) and glutamate pyruvate transaminase (GPT) (b). Experiments were conducted as described in Section 2. Values are mean ± standard error of the mean. Each group contained 12 animals. ^*∗∗*^ *p* < 0.01 and N.S. versus sham.

Plasma GOT and GPT activities were significantly augmented by 126% and 121%, respectively (^*∗∗*^ *p* < 0.01), 18 h after CLP (Figure 1, comparison between empty and black columns). The sepsis-induced increases in GOT/GPT activities returned to the control level following rhHsp72 administration (Figure 1, comparison between empty and shaded columns), demonstrating that rhHsp72 ameliorates liver function during sepsis progression.

### 3.2. Effects of rhHsp72 on Apoptosis Inhibition in Livers of Rats with Sepsis

**Figure 2:**
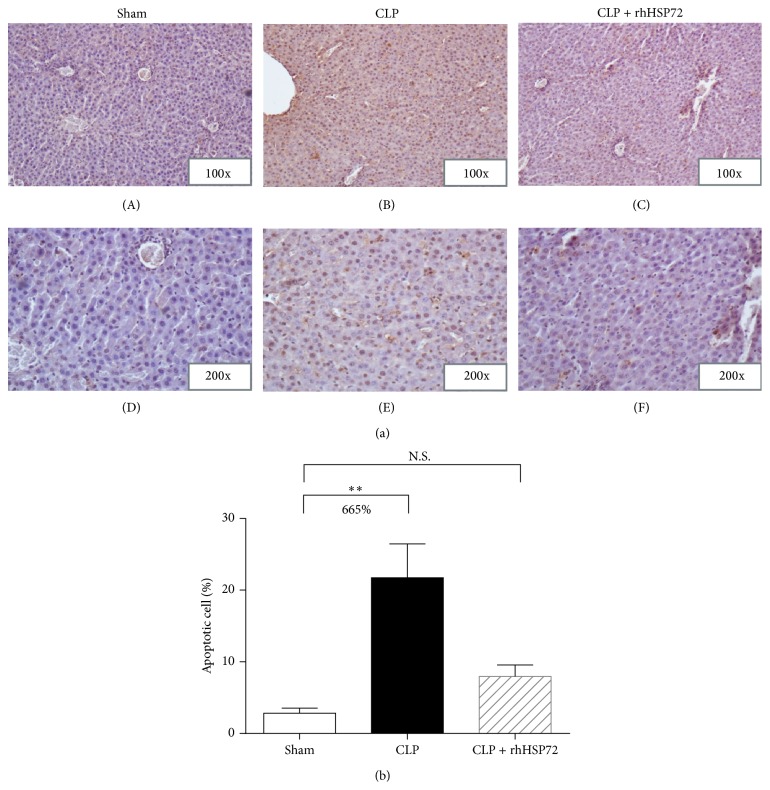
Effects of rhHsp72 on apoptosis inhibition in livers of rats with sepsis. Experiments were conducted as described in Section 2. Panel (a) plots the representative histograms of TUNEL and hematoxylin staining (sham: (A) and (D); CLP: (B) and (E); CLP+rhHsp72: (C) and (F)). Panel (b) depicts the quantitative analysis of TUNEL-/hematoxylin-positive cells. Values (means ± SE) presented in Panel (b) were obtained by dividing the number of TUNEL-positive cells by the total number of nucleoli. Each group contained 12 animals. ^*∗∗*^ *p* < 0.01 and N.S. versus sham.

The extent of sepsis-induced cell apoptosis was detected using TUNEL staining (Figure 2(a)). As shown in Figure 2(b), significant increases in the number of TUNEL-positive cells (21.7%) were observed in the CLP group. Sepsis increased the number of TUNEL-positive cells by 6.6 times (+665.7%) (Figure 2(b), comparison between black and empty columns), whereas the sepsis-induced augmentation in TUNEL-positive cells returned to the control level after rhHsp72 administration (Figure 2(b), comparison between empty and shaded columns; N.S.). These results indicate that exogenous Hsp72 protects liver cells from cell death during sepsis progression.

### 3.3. Effects of rhHsp72 on Bcl-2 and Bax Expression in Livers of Rats with Sepsis

**Figure 3:**
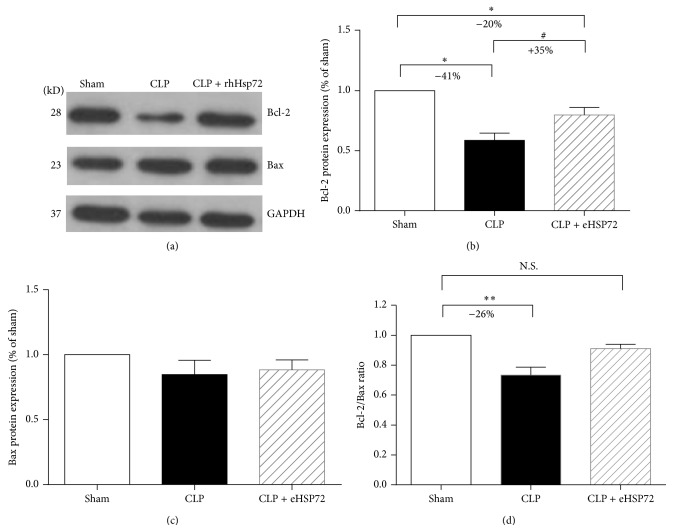
Effects of rhHsp72 on Bcl-2 and Bax expression in livers of rats with sepsis. Experiments were conducted as described in Section 2. Panel (a) is the Western blots showing the expression levels of Bcl-2, Bax, and GAPDH. Panels (b) and (c) present the intensity of the signals of Bcl-2 and Bax quantified using densitometry after normalization with that of GAPDH. Panel (d) represents the Bcl-2/Bax ratio. Values are mean ± standard error of the mean. Each group contained 12 animals. ^*∗*^ *p* < 0.05, ^*∗∗*^ *p* < 0.01 and N.S. versus sham; ^#^ *p* < 0.05; versus CLP.

To determine the effects of rhHsp72 on the modulation of apoptosis regulators, the protein levels of Bcl-2 and Bax in the liver were measured. As shown in Figure 3, Bcl-2 expression decreased by 41% (^*∗∗*^ *p* < 0.01) during sepsis (Figure 3(b), comparison between empty and black columns). The sepsis-induced decrease in Bcl-2 expression was reduced by 49% (^*∗*^ *p* < 0.05) after rhHsp72 administration (Figure 3(b), comparison between empty and shaded columns). The Bax protein level was unaffected during sepsis after rhHsp72 administration (Figure 3(c), comparison among empty, black, and shaded columns). Furthermore, the Bcl-2/Bax expression increased by 26% during sepsis, but the expression returned to the control level after rhHsp72 administration (Figure 3(d), comparison between empty and shaded columns; N.S.). Bcl-2 is an antiapoptosis mediator, whereas Bax is a proapoptosis mediator; therefore, the results suggest that rhHsp72 reduces liver apoptosis during sepsis progression.

### 3.4. Effects of rhHsp72 on Suppression of Apoptotic Mediator Activation

**Figure 4:**
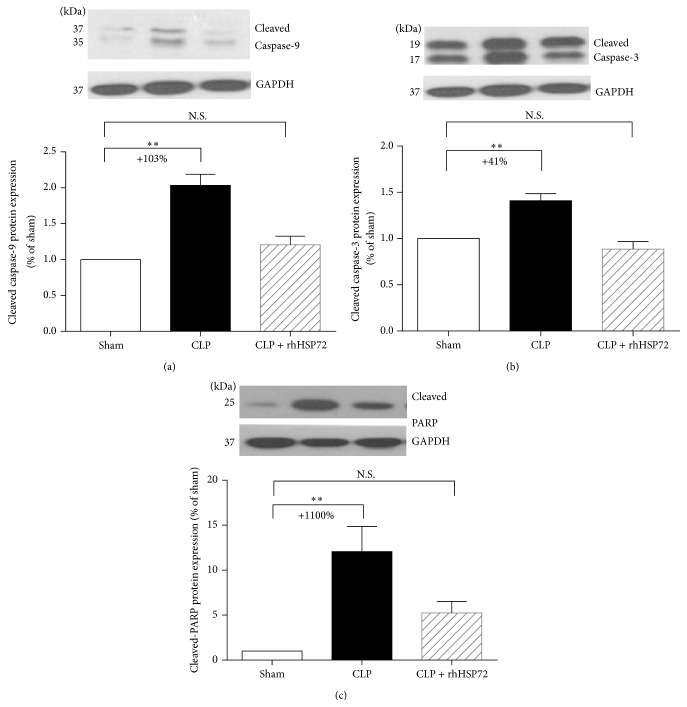
Effects of rhHsp72 on the suppression of apoptotic mediator activation. Experiments were conducted as described in Section 2. Values are mean ± standard error of the mean. Each group contained 12 animals. ^*∗∗*^ *p* < 0.01, and N.S. versus sham.

Caspase-3, a prominent signaling molecule in the apoptosis pathway, is initiated by caspase-9 in the mitochondria-dependent pathway. Activated caspase-3 cleaves PARP, which in turn promotes DNA degradation. The results in Figures 4(a) and 4(b) reveal that the expression of activated caspase-3 and caspase-9 increased during sepsis (comparison between empty and black columns; +103.3%; ^*∗∗*^ *p* < 0.001 and +40.9%; ^*∗∗*^ *p* < 0.001). The sepsis-induced increase in the activated capase-3 and caspase-9 levels returned to the control levels after rhHsp72 administration (Figures 4(a) and 4(b), comparison between empty and shaded columns). The results in Figure 4(c) confirm that the protein expression of cleaved PARP was upregulated (comparison between empty and black columns; +1106%; ^*∗∗*^ *p* < 0.001). The sepsis-induced upregulation of PARP expression returned to the control level after rhHsp72 administration (Figure 4(c), comparison between empty and shaded columns). These results suggest that the protective effects of rhHsp70 on liver cell apoptosis during sepsis progression are mediated through the suppression of apoptotic pathway mediators (i.e., activated caspase-3, activated caspase-9, and cleaved PARP).

## 4. Discussion

During sepsis, the liver plays a major role in the host defense mechanism [6, 32]. Liver dysfunction occurs frequently during sepsis (34%–46%) [7]. Attenuating hepatic apoptosis is critical in reversing sepsis-induced liver dysfunction [8, 33] and can lower the mortality rates in patients with sepsis [7]. Despite efforts to develop efficient antiseptic therapies, mortality of patients with sepsis remains high. Nevertheless, the results of a therapeutic strategy involving the use of exogenous Hsp72 to reduce sepsis-related mortality in animals are encouraging [34, 35]. However, the underlying mechanisms have not been explored.

Exogenous Hsp72 was found to accumulate mainly in the liver after intravenous and subcutaneous injections [27]. The present study demonstrates that rhHsp72 administration improves liver function and decreases hepatic apoptosis during sepsis (Figures 1 and 2). Factors contributing to liver dysfunction during sepsis involve uncontrolled systemic inflammation, hepatic ischemia, and deregulated cell apoptosis [6, 36, 37]. Apoptotic cell death is a key factor in the evolution of organ damage during sepsis. Jeschke et al. demonstrated that insulin improves hepatic integrity by decreasing hepatic cell death and inhibiting hepatic inflammatory responses in endotoxemic rats [5]. Moreover, blocking apoptosis improves the outcome in animals with severe sepsis [38, 39]. Based on these evidences, we suggest that exogenous Hsp72 possibly ameliorates liver dysfunction during sepsis by attenuating hepatic apoptosis.

The Bcl-2 family acts as a crucial checkpoint upstream of the mitochondrial apoptosis pathway [40, 41]. Bcl-2 is an antiapoptotic member of the Bcl-2 family, whereas Bax is a proapoptotic member [42]. Thus, the Bcl-2/Bax ratio is crucial in cell survival [40]. In this study, Bcl-2 expression was found to be upregulated, whereas Bax expression remained unaltered. Accordingly, the Bcl-2/Bax ratio appeared to increase in rats with sepsis in response to exogenous Hsp72. The caspase family is an executioner of apoptosis, in which caspase-3 is a crucial apoptotic protease in the final common pathway of the apoptotic cell death [43]. Mitochondrial proteins, such as cytochrome c, bind to Apaf-1 and pro-caspase-9 forming the apoptosome [44]. The apoptosome activates caspase-9 and initiates caspase-3 activation [45, 46]. The activated caspase-3 cleaves the endogenous substrates in the nucleus (PARP), leading to DNA fragmentation and eventually cell death [47]. Luo et al. reported that the protective effect of the exogenous Hsp72 against hydrogen peroxide-induced apoptosis arises from the attenuation of caspase-9 and caspase-3 activation in Schwann cells [24]. In this study, we found that the the Bcl-2/Bax ratio of liver was elevated in rats with the administration of exogenous Hsp72, associated with decreased protein expression of activated caspase-9 and caspase-3, and PARP cleavage. These results imply exogenous Hsp72 may prevent apoptosis of septic liver through inhibiting the mitochondrial-initiated caspase pathway.

## 5. Conclusions 

We demonstrate that treating sepsis with exogenous Hsp72 reverses liver dysfunction by downregulating hepatic apoptosis via the mitochondria-initiated caspase pathway. Our findings thus provide a biochemical basis for the use of rhHsp72 as a potential therapeutic agent in sepsis management.
